# Engineered Nanomaterials and Type I Allergic Hypersensitivity Reactions

**DOI:** 10.3389/fimmu.2020.00222

**Published:** 2020-02-14

**Authors:** Nasser B. Alsaleh, Jared M. Brown

**Affiliations:** ^1^Department of Pharmacology and Toxicology, College of Pharmacy, King Saud University, Riyadh, Saudi Arabia; ^2^Department of Pharmaceutical Sciences, Colorado Center for Nanomedicine and Nanosafety, Skaggs School of Pharmacy and Pharmaceutical Sciences, The University of Colorado Anschutz Medical Campus, Aurora, CO, United States

**Keywords:** nanoparticles, nanotechnology, nanosafety, allergy, anaphylaxis

## Abstract

Type I allergic hypersensitivity disorders (atopy) including asthma, atopic dermatitis, allergic rhinitis, and food allergy are on the rise in developed and developing countries. Engineered nanomaterials (ENMs) span a large spectrum of material compositions including carbonic, metals, polymers, lipid-based, proteins, and peptides and are being utilized in a wide range of industries including healthcare and pharmaceuticals, electronics, construction, and food industry, and yet, regulations for the use of ENMs in consumer products are largely lacking. Prior evidence has demonstrated the potential of ENMs to induce and/or aggravate type I allergic hypersensitivity responses. Furthermore, previous studies have shown that ENMs could directly interact with and activate key T-helper 2 (Th2) effector cell types (such as mast cells) and the complement system, which could result in pseudoallergic (non-IgE-mediated) hypersensitivity reactions. Nevertheless, the underlying molecular mechanisms of ENM-mediated induction and/or exacerbation of type I immune responses are poorly understood. In this review, we first highlight key examples of studies that have demonstrated inherent immunomodulatory properties of ENMs in the context of type I allergic hypersensitivity reactions, and most importantly, we attempt to put together the potential molecular mechanisms that could drive ENM-mediated stimulation and/or aggravation of type I allergic hypersensitivity responses.

## Introduction

### Background

The immune system is the primary system for host protection against pathogens and foreign substance. Functionally, it consists of the *innate* and *adaptive* immune systems. The body's physical (skin/epithelial layers) and chemical (antimicrobial proteins, mucus, normal flora, etc.) barriers are its first line of defense. Beyond this line resides cellular (e.g., macrophages, dendritic cells, neutrophils, mast cells, etc.) and non-cellular (e.g., the complement system, inflammatory mediators, etc.) components of the innate immune system. The adaptive (acquired) immune system is composed primarily of lymphocytes (i.e., T- and B-cells). The adaptive immune system is specific (antigen-specific) with a relatively slower response compared to the innate immune system and relies on optimal innate immune responses.

Antigen presentation is a key immunological process for the development of an adaptive immune response. Indeed, professional antigen presenting cells (APCs) such as dendritic cells and macrophages are critical for optimal adaptive immune responses. Aberration in the function of the innate or adaptive immune system could be associated with various pathological outcomes in the context of immune activation, suppression, or modulation. Importantly, overstimulation of the immune system could result in detrimental consequences, known collectively as *immune hypersensitivity reactions*. These reactions are typically triggered by harmful pathogens, pathogenic products as well as innocuous antigens. Immune hypersensitivity reactions are classified into 4 types according to the Gell Coombs classification. These are type I (immediate, IgE-mediated), type II (cytotoxic, IgG- and IgM-mediated), type III (antigen-antibody complexes), and type VI (delayed, T cell-mediated). The term “allergy” is loosely used by both the lay as well as the scientific communities to indicate all types of immune hypersensitivity reactions (mentioned above). However, according to the American Academy of Allergy, Asthma and Immunology (AAAAI), the term allergy should rather be used to define type I (immediate) hypersensitivity immune reactions (atopy or atopic allergy) to “ordinarily harmless substance” (1). In this review, we focus our discussion on the influence of ENMs on type I allergic (atopic, IgE-mediated) immune responses. We also discuss potential mechanisms of ENM-mediated allergic and allergic-like (pseudoallergic, a form of allergic responses that are not driven by IgE and does not require prior sensitization to an allergen) immune responses (2).

### Type I Allergic Hypersensitivity and Atopic Disease: Molecular Mechanisms of Disease

Atopic diseases are increasing in developed and developing countries reaching epidemic levels (2, 3). It is estimated that 20–30% of the population worldwide suffers from allergic disease (4). According to World Health Organization (WHO), about 300 million people have asthma with a negative impact on quality of life of individuals, families, and societies (4). Development of atopy is a complex, combinatorial process, which is not only due to environmental exposures (i.e., allergens), but also genetic factors (5). Allergic diseases include asthma, food allergy, allergic rhinitis, allergic conjunctivitis, atopic dermatitis, atopic eczema, and life-threatening anaphylaxis.

Allergic form of atopic disease develops following expansion of T helper 2 (Th2) cells and immunoglobulin (Ig) class switching to IgE by plasma cells following first exposure to an allergen (e.g., peanut, dust mite, mold, animal dander, pollen, insect stings, etc.). Allergens (antigens) are phagocytosed by antigen presenting cells (APCs) including dendritic cells (DCs) and macrophages typically at the periphery, which could result in cell maturation, activation, and migration to the lymphatic system. At a secondary lymphoid organ (e.g., lymph node, peyer's patches, etc.), APCs present parts of processed antigens on major histocompatibility complex (MHC-II), and under optimal conditions (e.g., affinity of antigen binding to T-cell receptors, co-stimulatory signals, cytokine milieu, etc.), this leads to expansion of clonal T-cells. IgG and subsequently IgE are then produced by T-cell-regulated plasma cells (mature phenotype of B-cells). IgE binds to the high affinity IgE receptor (FcεR1) primarily on basophils (circulation) and mast cells (tissues). This is known as the *sensitization phase*. Upon a second exposure to the same allergen (antigen), binding of the allergen to IgE-bound FcεR1 on the pre-sensitized Th2 effector cells including basophils and mast cells results in crosslinking of FcεR1 and consequent (immediate) release of a plethora of preformed mediators including vasoactive molecules such as histamine and heparin as well as a number of proteases such as tryptase and chymase. Activated cells also synthesize other inflammatory mediators including *de novo* lipid mediators including prostaglandins and leukotrienes as well as Th2-type cytokines and chemokines such as IL-2, IL-4, IL-5, IL-10, IL-13, and TGFβ. Together, these mediators result in the initiation and development of the underlying pathological outcomes of type I allergic (atopic) disease. Typically, these outcomes are manifested by local and/or systemic classic type I allergic symptoms including sneezing, runny nose, watery eyes, cutaneous weal and flare, bronchoconstriction, GI disturbance and sometimes and life-threatening anaphylaxis. Further exposure to the same allergen may lead to aggravated allergic symptoms due to subsequent clonal expansion of T-cells along with the recruitment of other types of effector immune cells (e.g., eosinophils).

### Engineered Nanomaterials and the Immune System

**Figure 1:**
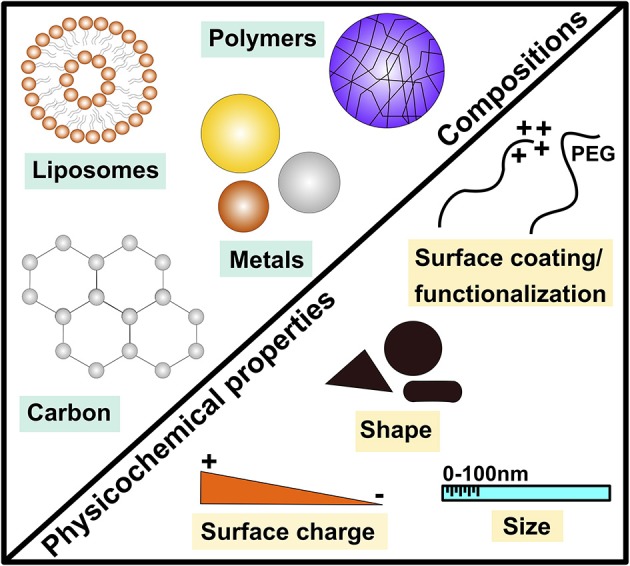
Examples of major engineered nanomaterial compositions and key physicochemical properties. This demonstrates the diverse nature of engineered nanomaterials (ENMs) by highlighting examples of major materials (compositions) of ENMs including metals, carbonic, polymeric and liposome-based ENMs as well as key physicochemical properties such as size, shape and surface coating/functionalization.

Nanotechnology is one of the major innovations in the twenty-first century and comprises one of the technological developments associated with the fourth industrial revolution. There has been large institutional support worldwide over the past couple of decades toward basic research as well as development and utilization of nanotechnologies for many applications and across multiple industries (6). ENMs are materials at a size range of 1 to 100 nanometers in at least one dimension. They are made with extremely precise physicochemical properties (e.g., size, shape, surface charge, crystallinity, etc.) (Figure 1). Today, nano-enabled materials are widely utilized in consumer and medical products and the number of ENM-enabled applications are increasing everyday across a broad range of industries including healthcare, food, textiles and electronics. For instance, in the biomedical fields, ENMs are being utilized for the assembly of complex (functionalized) drug platforms for imaging, targeted delivery and controlled release all within the same nanoplatform (7). Other ENMs have been developed for their antimicrobial properties and are being investigated for the use in cases of microbial resistant (8). Such large utilization of ENMs including in biotechnology and biomedicine, particularly under minimal regulation (largely due to uncertainty regarding exposure and hazard of ever-emerging ENMs), is of concern and could be problematic. Indeed, a growing body of evidence suggests a potential for novel nano-specific toxicity of ENMs (9). Although there have been efforts including specific research programs and consortia across countries and continents to address health and environmental safety of ENMs, our molecular understanding about ENM-mediated bio-physicochemical interactions at the nano-bio interface remains very limited (10). It is noteworthy that due to their unique physicochemical properties (compared to their bulk counterparts), ENMs have shown wide biodistribution and tissue accumulation (bio-kinetics). This “novel” bio-kinetics could contribute to their novel biological (toxicological) outcomes. Such wide biodistribution and access to tissue locations which would have not been possible with bulk materials, could indeed be problematic particularly from an allergic point of view (e.g., direct interaction with Th2-type effector cells such as mast cells, basophils and eosinophils).

Accumulating evidence has revealed that the immune system is a major target system for ENM-mediated toxicological outcomes (11, 12). This is unsurprising considering the vital role of the immune system in host protection against environmental exposures. Importantly, one of the manifestations of the immune system response to environmental exposures is type I allergic hypersensitivity which, as mentioned above, could result in serious consequences such as anaphylaxis. In this concise review, we discuss the current state-of-art research on the influence of ENMs to modulate immune responses in the context of type I allergic hypersensitivity. Specifically, we present and discuss examples of key studies that have demonstrated inherent immunomodulatory properties of ENMs, which could be implicated in allergic and non-allergic atopic disease. Furthermore, we discuss later in this review the potential molecular mechanisms of ENM-induced immunomodulation that could contribute to type I allergic hypersensitivity. This is by no means a comprehensive review of ENM immunomodulatory properties and excellent detailed reviews can be found elsewhere (13, 14).

## Engineered Nanomaterials and Type I Hypersensitivity

### The Feraheme® Case

Ferumoxytol (Feraheme®) is a carbohydrate-coated superparamagnetic iron oxide (SPIO) nanoparticle-based drug given intravenously for iron replacement to hospitalized patients with chronic kidney disease (15). Recently, treatment with ferumoxytol resulted in severe anaphylactic reactions, 18 of which were fatal (16). As a result, the FDA strengthened warning of serious allergic responses to ferumoxytol including changes in prescribing instructions (black box warning) such as slow infusion of the drug, the presence of healthcare personnel while giving the drug and immediate availability of therapies for the treatment of anaphylaxis and hypersensitivity. Importantly, the underlying mechanism of ferumoxytol-induced fatal anaphylaxis is completely unknown including whether the drug worked as a hapten or allergen to those who had reaction to the drug (i.e., susceptible population). It is worth mentioning that SPIO nanoparticle-based medications are widely utilized for imaging applications as contrast agents for MRI and have been shown to be associated with allergic-like (pseudoallergic) reactions (17, 18). The Feraheme® incident raises a red flag for the significant impact of type I hypersensitivity reactions that could result from exposure to ENMs and the urgency of understanding ENM-associated allergenicity in future nano-immunosafety studies. It is noteworthy that other liposomal, micellar and polymeric nano-formulations have been previously shown to be associated with hypersensitivity reactions (19).

### Poor Inherent Immunogenicity of ENMs

Because of their small size, most ENMs are considered poorly immunogenic even in the presence of strong adjuvants in terms of inducing an adaptive immune response-mediated production of antibodies (20). However, it has been shown that when ENMs such as liposomes, synthetic polymers, and fullerenes are conjugated with a protein carrier or polymer, specific antibodies were produced against ENMs (21–23). The introduction of ENMs even those that have no biomolecular conjugates into biological environments (e.g., blood, airways, GI tract, etc.) is going to form an ENM biocorona almost instantly which can be composed of different macromolecules (e.g., albumin, surfactant, etc.). Importantly, current and future nanotherapeutics are being developed as sophisticated systems that constitute multiple targeting and stabilizing macromolecules including proteins and hence production of antibodies to these systems is likely (there are certainly ways to mask or reduce the recognition of such complex nanotherapeutic systems by the immune system e.g., use of polymers, which could modulate the adsorption of plasma proteins leading to “stealth effect”; however, this topic is beyond the scope of this review) (24). Accordingly, potential immunogenicity of ENMs, particularly those that are composed of multiple compositions and biomolecules, should always be taken into consideration during toxicological testing to ensure safety of future nanomaterials.

It is worth noting that to date there is limited evidence of ENM-mediated isotype class switching to IgE. One report has previously shown that repeated exposure to silver nanoparticles (AgNPs) at high doses resulted in increased serum IgE levels (25). However, further studies are warranted to determine whether IgE class switching could happen due to inherent properties of ENMs.

### Evidence of ENM-Mediated Activation and Exacerbation of Type I Allergic Responses

Accumulating evidence has demonstrated that exposure to ENMs including carbon nanotubes (CNTs), titanium dioxide (TiO_2_), gold (AuNPs), silver (AgNPs), silica and zinc oxide (ZnO) nanoparticles, alongside Th2-type allergens (e.g., ovalbumin) or in experimental animal models of type I allergy (e.g., atopic dermatitis, AD) resulted in exacerbation of allergic hypersensitivity responses (26–31). For example, it has been shown that co-administration (intradermal injection) of amorphous silica nanoparticles with dermatophagoides pteronyssinus (Dp, a type of mites) in NC/Nga mice (an inbred mouse model of that develops skin lesions similar to those seen in human AD), has resulted in aggravation of AD symptoms including elevated serum IgE levels and systemic Th2 response (28). Furthermore, the authors have found that such response was inversely correlated with particle size. It has also been shown in a subsequent study by the same group that cutaneous exposure to agglomerates of silica nanoparticles (30 nm in diameter) and mite allergen resulted in a low IgG/IgE ratio and increased sensitivity to anaphylaxis (32). Another report has demonstrated that repeated topical exposure to ZnO nanoparticles (but not their bulk counterparts) in a BALB/c mouse model of AD, although suppressed local inflammation, resulted in aggravated response to the allergen (ovalbumin/staphylococcal enterotoxin B), which was manifested by elevated serum IgE levels suggesting a possible nanoparticle-specific role in priming Th2-type allergic responses (30). In a mouse model of asthma (toluene diisocyanate, TDI-induced asthma), pulmonary exposure to TiO_2_ nanoparticles and AuNPs worsened pulmonary inflammation including edema, airway hyperreactivity and infiltration of inflammatory effector immune cells (29). In a study assessing potential allergenicity of AgNPs in a healthy and allergic mouse model (ovalbumin-induced) through pulmonary (inhalation) exposure, it has been shown that exposure to AgNPs for 7 days (6 h/day) resulted in an inflammatory allergic response including increase in serum ovalbumin-specific IgE levels, IL-13 levels and inflammatory cell count, airway hyperreactivity and accumulation of AgNPs in lungs of both the healthy and allergic mouse groups (26). In a mouse model of AD, it has been shown that co-administration of AgNPs resulted in aggravated skin lesions, mast cell infiltration and increased serum IgE levels (33). Such response was only manifested with 5 nm AgNPs (vs. 100 nm AgNPs), which could be attributed to the large surface area and/or faster release of silver ions. Taken together, these findings suggest the potential adjuvancy properties of ENMs toward type I allergic hypersensitivity responses. To date, the underlying molecular mechanisms of these findings are largely lacking. Later in this review, we discuss potential molecular mechanisms that could drive such observed ENM-mediated aggravation of type I allergic and allergic-like (pseudoallergic) responses (e.g., mediated by activation of the complement system, Th2-type effector cells, etc.). It is also important to note that a number of different ENMs in the aforementioned studies are considered toxicologically inert to a large extent and thus are widely utilized or are under investigation for novel applications including TiO_2_ and ZnO ENMs. Therefore, it is important to note that absence of direct ENM-mediated toxicity and/or immunogenicity do not necessarily imply lack of adverse health outcomes emphasizing the importance of the context (disease model, co-exposure, susceptible population, etc.).

**Figure 2:**
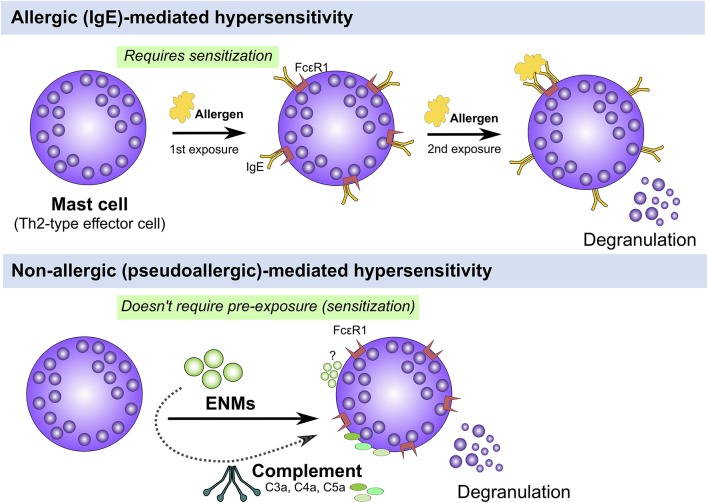
IgE (allergic) vs. non-IgE (pseudoallergic)-mediated activation of mast cells. Mast cells can be activated either through an IgE or non-IgE pathways. IgE stimulation requires pre-exposure to an antigen (e.g., pollen, mite, etc.) and upon a 2nd exposure, IgE-bound FcεR1 are crosslinked leading to activation of signal transduction pathways that culminate in cell degranulation and activation **(Top panel)**. Mast cells can be also activated through non-IgE pathways by a wide range of materials (e.g., cytokines, complement fragments, basic polypeptides, environmental toxicants, toxic venoms, etc) which don't require prior exposure and may lead to mast cell degranulation and/or activation **(Bottom panel)**. The underlying molecular mechanisms of non-IgE-mediated mast cell degranulation and activation are largely unknown.

Atopy and atopic disease can be driven by non-IgE (also called non-allergic, pseudoallergic, and intrinsic)-mediated mechanisms, that is, patients could develop atopic disease-related symptoms with no elevation in levels of serum IgE (2). A growing body of research has demonstrated potential direct activation of the complement system and non-IgE mediated activation of effector immune cells including mast cells and basophils that could lead to type I hypersensitivities (Figure 2). However, the underlying mechanisms are poorly documented. Mast cells are key tissue-resident effector cells that are present in large numbers in locations with close proximity to the external environment, such as the skin and mucosa, acting as sentinels against pathogens and environmental insults armed with secretory granules and always ready to respond to insult (34). Indeed, mast cells play key roles as a first line of defense (of the innate immune system) with their multi-faceted capabilities in sensing a multitude of environmental exposures and danger signals with their diverse preformed granular content leading to rapid recruitment and activation of other immune cells including neutrophils and dendritic cells (34). Therefore, it is reasonable to speculate that some ENM-mediated adverse responses could be driven by direct or indirect (e.g., the complement system) activation of mast cells, which results in allergic-like responses, worsened inflammatory reactions and even serious anaphylactic reactions as the case of ferumoxytol. Indeed, we and others have previously demonstrated a role for mast cells in ENM-mediated toxicological outcomes *in vivo*. For instance, we have shown that exposure (inhalation) to cerium oxide (CeO_2_) nanoparticle in C57BL/6 mice resulted in local pulmonary inflammation as well as systemic cardiovascular complications (35). The pulmonary inflammation was largely attenuated and the cardiovascular responses were absent in a mast cell-deficient mouse model (B6.Cg-KitW-sh mice) suggesting a role for mast cells in driving ENM-mediated adverse responses (35). Similarly, toxicological outcomes including pulmonary and cardiovascular adverse responses following aspiration of multiwalled carbon nanotubes (MWCNTs) were absent in mast cell-deficient mice (B6.Cg-KitW-sh) (36). In Sprague-Dawley rats, it has been demonstrated that intragastric exposure to TiO_2_ nanoparticles once daily for 30 consecutive days resulted in increased numbers of mast cells in stomach tissue particularly in the 3-week old rats compared to the 8-week old rats (37). The authors have shown that there was no change in the level of serum IgE and histamine suggesting a potential non-IgE mediated activation of mast cells (37). Intravenous single-dose exposure to amorphous silica nanoparticles in Wistar rats resulted in increased mast cell numbers, liver tissue remodeling and fibrosis 30 days post exposure (38). Together, these results suggest a potential non-IgE mast cell involvement in the toxicological outcomes of ENMs of different materials and physicochemical properties. Thus, understanding ENM direct interaction with mast cells at the molecular level could provide some insights into the potential underlying molecular mechanisms of non-IgE-mediated allergic responses following exposure to ENMs.

## Potential Mechanisms of Enm-Induced Triggering and/or Exacerbation of Type I Allergic and Allergic-Like Responses

Currently, there is a major gap in knowledge in regard to our understanding of the underlying cellular and molecular mechanisms that drive ENM-induced activation and/or exacerbation of allergic and allergic-like (pseudoallergic) responses. Nevertheless, there have been attempts to elucidate the underlying mechanisms and here we review key examples. We also discuss other relevant mechanisms that are studied in other contexts (e.g., inflammasome), which could contribute to allergic and allergic-like responses.

**Figure 3:**
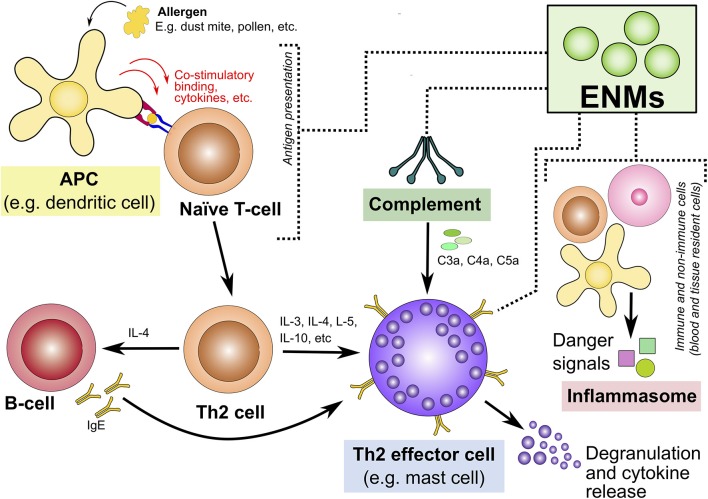
Potential mechanisms underlying engineered nanomaterial-mediated induction and/or exacerbation of type I allergic hypersensitivity reactions. This highlights four potential mechanisms that could explain ENM-induced allergic hypersensitivity reactions. These are highlighted in colored boxes: interaction with/activation of the (1) complement system, (2) inflammasome activation, (3) APCs, and (4) Th2-type effector cells. ENM, engineered nanomaterials; APCs, antigen presenting cells; Th2, T-helper-2; IgE, immunoglobulin E; C3a, complement component 3a; C4a, complement component 4a; C5a, complement component 5a.

The following section discusses 4 potential mechanisms that could contribute to ENM-mediated induction and/or exacerbation of type I allergic hypersensitivity reactions (Figure 3). These include the influence of ENMs on the complement system, inflammasome activation, APCs and key Th2 effector cells.

### Interaction With and Activation of the Complement System

The complement system is composed of a number of soluble proteins that become activated in an enzymatic cascade in response to invading pathogens and foreign substance. It is one of the basic conserved innate immune responses that help eliminate invading pathogens and foreign substance. The complement system can be activated via 3 pathways, namely, classical, alternative, and lectin pathways. These pathways differ in the molecules they recognize; however, they all converge into cleaving a central complement protein, C3. Four outcomes in response to complement activation may occur including pathogen opsonization (tagging for recognition) and lysis (forming membrane pores) as well as cellular chemotaxis and anaphylaxis. The latter particularly is of critical significance since it attracts and activates Th-2 type effector cells such as basophils and mast cells resulting in allergic-like responses. Release of cellular mediators (i.e., cell degranulation) by these Th2 effector cells leads to a myriad of biological effects locally and/or systematically ranging from mild allergic responses to serious anaphylaxis. Importantly, ENMs of different compositions and physicochemical properties, including clinically-approved liposome-based and micellar drugs, have been shown to activate the complement system through its different pathways leading to pseudoallergic responses (also called complement activation-related pseudoallergy, CARPA) (39–41). Such pseudoallergic response or hypersensitivity reaction (HSR) can be manifested by a range of mild to severe symptoms including hypotension, bronchospasm and dyspnea, GI disturbance and skin flushing. Interestingly, it has been previously demonstrated that even subtle variation in ENM physicochemical properties of clinically-approved liposome-based drugs (also referred to as non-biological complex drugs or nano-similars) such as Doxil® and Caelyx® or even batch-to-batch variability can modulate complement responses (42). Another class of nanomedicines that have been associated with allergic-like reactions are superparamagnetic iron oxide (SPIO) nanoparticles-based medications, which are widely utilized for MRI as contrast agent including Resosvist® and Sinerem® (43, 44). Despite this recognition of pseudoallergic reactions, the underlying molecular mechanisms are still to be unraveled. Key physicochemical properties of ENMs including particle size, morphology and surface properties may play crucial roles in activating the complement system (45). Due to the large surface area of ENMs, proteins and other macromolecules readily bind/adsorb to ENM surfaces forming a biocorona, which is highly dynamic. Importantly, ENM biocorona has been shown to play a major role in ENM-induced complement activation (46). Indeed, one proposed mechanism of ENM-induced complement activation and C3 opsonization is attributed to subsequent conformational changes of ENM-adsorbed biomacromolecules (i.e., exposing antigenic epitopes) (46). Further, it has been recently shown that natural antibodies play a key role in ENM-induced complement opsonization (47). Importantly, such mechanisms appears to be universal to multiple pre-clinical and clinical nanomedicines (47). Taken together, due to ENM size and physicochemical properties (e.g., shape, surface charge, topography, hydrophobicity, etc.), which could resemble nano-sized pathogens such as viruses, as well as large surface area which leads to almost instant formation of a biocorona once ENMs are in contact with biological media, routine assessment of complement activation is deemed necessary. Moreover, further research and emphasis on understanding ENM-bio interactions with the complement system at the molecular level is key for designing ENMs that lack undesired pseudoallergic responses.

### Release of “Danger Signals” and Activation of the Inflammasome

The innate immune system has long evolved to sense and recognize pathogenic and foreign molecules. For example, innate immune cells such as granulocytes and macrophages can recognize pathogen-associated molecular patterns (PAMPs) such as lipopolysaccharide and double-stranded RNA. Additionally, they can recognize danger/damage molecular patterns (DAMPs), which result from cellular insult/damage and are released into the extracellular space. Examples include heat shock proteins, HMGB1 (a chromatin-associated protein) and uric acid. Activation of the inflammasome in the case of DAMPs leads to a sterile inflammatory response. PAMPs and DAMPs are recognized by pattern recognition receptors (PRRs) such as Toll-like receptors (TLR) and NOD-like receptors (NLR). The inflammasome is a large cytosolic multiprotein complex that is assembled in response to NLR activation (e.g., NLRP1 and NLRP3) to process and produce pro-inflammatory cytokines such as IL-1 and IL-18 and/or induce cell death (pyroptosis) (48). The inflammasome is implicated in a wide range of inflammatory diseases and cancers (48). NLRP3, one of the most commonly studied member of the NLR family, recognizes a wide range of molecules of self (e.g., extracellular ATP, amyloid β and hyaluron) and non-self origen (e.g., alum, asbestos, UV-radiation, bacterial toxins and double-stranded RNA).

Importantly, ENMs of different compositions (e.g., carbon, metal, polymers, etc.) and physicochemical properties (e.g., size, shape, surface functionalization, etc.) have been shown to activate the inflammasome. Furthermore, ENMs of different compositions and physicochemical properties, have been shown to induce inflammasome formation through multiple mechanisms including frustrated phagocytosis, potassium efflux, oxidative stress, lysosomal damage, and cathespins release (49). For instance, it has been shown that similar to asbestos, long, needle-like carbon nanotubes (CNTs), but not short CNT, long-tangled CNTs or carbon black, activated the NLRP3 inflammasome in LPS-primed primary human macrophages, which appeared to be dependent on multiple mechanisms including formation of reactive oxygen species, P2X7 receptor, cathepsin B activity as well as and Src/Syk tyrosine kinases activity (50). It has been shown in a recent study that shape of ENMs is a key factor in the activation of the inflammasome (51). Specifically, using 4 different shapes of iron oxide nanoparticles (IONPs), the authors have demonstrated that exposure to the ocatpod and plate-shaped IONPs was associated with increased release of IL-1β release and more cell pyroptosis when compared to the sphere and cube nanoparticles. It has been also found in the same study that activation of the inflammasome by the iron oxide nanoparticles was only partially mediated by NLRP3 (51). Such findings indicate that the same ENMs may induce activation of the inflammasome through multiple receptors/biological targets. Although such inherent ability of ENMs to induce an inflammatory response through activation of the inflammasome in the context of type I allergic hypersensitivity would be problematic, other contexts such as in the case of vaccination would be desired. In either case, better understanding of the structure-activity relationship and the underlying molecular mechanisms by which ENMs trigger activation of the inflammsome is of critical importance for developing future nanomedicines that are either devoid of inflammasome activation such as in the case of hypersensitivity and autoimmunity or that are deliberately designed to activate the inflammasome such as in the case of immune system stimulation (e.g., vaccination and cancer immunotherapy).

It is also critical to mention here that integrity of the epithelial barrier is a key component in the development of allergic disease. Loss of epithelial barrier integrity has been shown in a wide range of type I allergic hypersensitivity conditions and typically it precedes full development of allergic disease (52). Importantly, ENMs have been previously shown to damage the epithelial barrier (pulmonary and intestinal mucosa, skin, etc.), not only resulting in the release of danger signals/DAMPs, activation of the inflammasome and recruitment of immune cells, but could potentially give access to allergenic pathogens and pathogenic components as well as environmental pollutants [reviewed in more detail in (53)]. Such events are greatly influenced by type (composition) and physiochemical properties of ENMs including large surface (reactive) area and ratio aspects of ENMs (54). Some of these properties have been widely established in the field of particulate toxicology and the association between environmental pollutants and type I allergic disease is well-recognized (55). Currently, there is a lack of comprehensive *in vivo* studies in the area of research (i.e., toxicity of nanomaterials on epithelial barriers) and further studies are warranted particularly in the context of type I hypersensitivity.

### Interaction With Antigen Presenting Cells (APCs)

Professional APCs such as dendritic cells (DCs) and macrophages play a key role in the interplay between innate and adaptive immunity (56, 57). APCs are critical for optimal development of Th2 adaptive immune responses (58). They are also crucial for the development of immune tolerance to innocuous pathogens and foreign substance (59, 60). Therefore, optimal functioning of APCs is of critical importance for the homeostasis of the immune system and a fine line exists between immunity and tolerance (regulated by a complex set of factors including nature of antigen, cytokine milieu, and binding of co-stimulatory molecules). Accordingly, manipulation of the function of APCs can be associated with detrimental consequences manifested by a wide range of pathological conditions such as autoimmune disease, allograft rejection, and cancer (56, 57). To this end, a number of studies have assessed direct interactions between ENMs and APCs. It has been elegantly demonstrated that ENMs can traffic to the draining lymph nodes targeting resident DCs and macrophages, a response that has been shown to be inversely correlated with the size of ENMs (61, 62). Such findings are of key importance demonstrating ENMs improved capability (vs. their bulk counterparts) to reach and directly interact with APCs with a potential to modulate and compromise normal and optimal cellular functions. Indeed, previous evidence has demonstrated the capacity of ENMs to modulate the function of APCs including perturbation of their polarity which is critical particularly in the context of inflammatory disease and type I allergic hypersensitivity reactions (63–65).

It is noteworthy that there has been an extensive amount of research on the use of ENMs as adjuvants and delivery systems for vaccines (nanovaccinology) or immunotherapy. However, most of this work has utilized ENMs (and larger particles, usually up to 1 micrometer in diameter/dimensions) for the primary purpose of targeting and/or activation of specific subsets of immune cells including APCs (i.e., desirable immunostimulatory properties) (66). Such studies were not concerned with understanding if and how ENMs and their inherent properties influence cell function at the cellular and molecular levels. On the other hand, there exist other studies in the literature that have investigated ENMs influence on APC activation, maturation and modulation of their function including antigen response. For instance, it has been previously demonstrated that *in vitro* exposure of bone marrow-derived DCs (BMDCs) to carbon black (CB) nanoparticles for 24 h resulted in activation of BMDCs based on surface expression of CD80, CD86, and MHC-II as well as led to enhanced response of allogeneic mixed lymphocyte reaction (MLR), a measure of T-cell proliferation (67). Similar findings have also been demonstrated in response to TiO_2_ and amorphous silica (SiO_2_) nanoparticles, which are widely utilized across multiple industries including the food industry. Specifically, exposure to ENMs resulted in activation of bone BMDCs including upregulation of MHC-II, CD80, and CD86 and the inflammasome (68). These reports suggest a potential priming effect on adaptive immunity following exposure to ENMs and depending on the context of exposure and nature of antigen involved, ENMs could prime the immune system toward either Th1 or Th2 phenotype. This has been shown before where intranasal co-exposure to ovalbumin with CB or TiO_2_ nanoparticles resulted airway inflammation and augmented antigen-mediated response including an amplified release of Th2-type cytokines including IL-4, IL-5, IL-10, and IL-13 (27). Such airway sensitization has been also shown in a mouse model of diisocyanate-induced asthma in response to TiO2 nanoparticle exposure (29).

Differentiation of naive T-cells is a complex process and involves multiple factors including nature of antigen, signals for APC maturation, cytokine milieu, and co-stimulatory binding. To date, the exact mechanisms of T-cell differentiation and maturation into specific phenotypes are yet to be elucidated. Interestingly, it has been previously shown that ENMs could prime APCs toward either Th1 or Th2 phenotype depending on the redox potential of ENMs (69). Specifically, it has been demonstrated *in vitro* that exposure to oxidant TiO_2_ nanoparticles primed primary human monocyte-derived DCs toward a Th1 phenotype whereas antioxidant cerium oxide (CeO_2_) nanoparticles primed the same cells toward a Th2 phenotype. This is of critical interest particularly that the observed priming response of DCs was not influenced by the nature of the antigen (i.e., toward a predetermined Th1 or Th2 phenotype) but rather the inherent properties of ENMs. These data suggest ENMs could drive or prime adaptive immune responses toward distinct phenotypes. Nevertheless, these findings are yet to be demonstrated in *in vivo* settings.

Taken together, from the aforementioned examples one could speculate that a plausible mechanism by which ENMs induce or exacerbate allergic responses is through direct interaction with and modulation of APC function (e.g., maturation/polarization, activation, modulation of key signaling events during antigen presentation, etc.). Due to the critical role of APCs in the development of optimal adaptive immune response, further mechanistic work is highly warranted in this area to gain insights into the underlying molecular mechanisms through which ENMs influence APC function or modulate key molecular events during antigen processing and presentation. Indeed, we believe that understanding ENM-mediated immunomodulatory properties at the APC level is going to be key in the discovery of novel nanotherapeutics for a wide range of pathological conditions not only in the context of immune system disease (e.g., autoimmune diseases, allergic hypersensitivity reactions) but also extend to other pathological conditions such as cancer, metabolic syndrome and neuroinflammatory disease.

### Interaction With Key Th-2 Effector Immune Cells Including Mast Cells, Basophils, and Eosinophils

As mentioned earlier in this review, mast cells are a key Th2 effector cell type that play prominent roles in atopic disease. Importantly, mast cells have been shown to be involved in ENM-induced toxicological outcomes of different ENM compositions and physicochemical properties. Accumulating evidence has demonstrated that ENMs can induce non-IgE-mediated mast cell activation in response to a wide range of ENM compositions including different metal and metal oxide ENMs such as AuNPs, AgNPs, copper oxide (CuO), SiO_2_, and TiO_2_ (70–72) (Figure 2). However, at the cellular and molecular levels, little is understood regarding the underlying mechanisms of ENM-induced mast cell activation. Over the past few years, there have been attempts to understand and elucidate the underlying molecular mechanisms of ENM-induced mast cell activation. Both basophils and mast cells are capable of sensing a wide range of environmental cues such as PAMPs and DAMPs (also known as danger signals and alarmins). Interleukin (IL)-33 is a member of the IL-1 family and a ligand for ST2 receptor. IL-33 is considered a novel alarmin that acts as an endogenous danger signal released by damaged epithelium/endothelium as a result of trauma or infection (73). Its receptor (the ST2 receptor) is highly expressed in Th2-type cells including in the mast cell and has been previously shown to be associated with Th2-type inflammatory responses such as in asthma and rheumatoid arthritis (74). We have previously demonstrated a role for the mast cell IL-33/ST2 axis in response to MWCNT toxicity (36). Specifically, using mast cell-deficient and ST2^−/−^ mouse models, we have demonstrated that MWCNT-induced adverse pulmonary and cardiovascular responses were almost completely abolished in the absence of mast cells or lack of expression of the mast cell ST2 receptors (36). Based on these findings, a novel mechanism has been proposed for MWCNT-mediated toxicity whereby pulmonary exposure to MWCNTs results in damaged lining epithelium of the airways and lungs which in turn results in the release of IL-33 that subsequently activate mast cells to release a myriad of inflammatory mediators.

*In vitro* exposure of RBL-2H3 cells (a rat basophilic leukemia cell line) to a mixture of anatase and rutile TiO_2_ nanoparticles has been shown to result in a concentration-dependent degranulation of mast cells and histamine release (75). The TiO_2_ nanoparticle-induced histamine release was calcium-dependent and involved the phospholipase C (PLC)/inositol triphosphate (IP3)/endoplasmic reticulum pathway, which was (at least partially) mediated by the L-type calcium channels but also through TiO_2_ nanoparticle-mediated disruption of cell membrane (75). The authors have argued that TiO_2_ nanoparticle-mediated generation of reactive oxygen species (ROS) was a major driving factor in cell activation and release of mediators. Our group has demonstrated that *in vitro* exposure of mouse bone marrow-derived mast cells (BMMCs) to AgNPs led to robust mast cell degranulation potentially through membrane interaction leading to activation of cell signaling pathways including PLCγ, PI3K, and PKC and influx of calcium, which was largely mediated by the calcium release activated channel (CRAC) channels (76). In another study, we have shown ROS generation in BMMCs in response to AgNP exposure, however, whether the ROS generation is the driving mechanism for AgNP-induced mast cell activation or only a mediator in cell activation response is yet to be determined (77). It is worth noting that ROS generation in mast cells has been well-established following an allergen challenge (in response to FcεR1 receptor activation), however, whether FcεR1-mediated ROS generation is indispensable for allergen-mediated mast cell activation is still debated (78). In an effort to understand potential novel non-IgE-mediated mechanisms of mast cell activation, we carried out an integrated transcriptomics (RNAseq analysis) and gene wide association (GWA) study, which has demonstrated gene susceptibility in response to AgNP-induced degranulation of BMMCs among a panel of mouse strains (71). Our transcriptomic analysis suggested involvement of multiple molecular targets including thioredoxin-interacting protein (Txnip) and a number of signaling components related to G-protein coupled receptors (GPCRs), some of which are currently under investigation (71). This is of particular interest considering previously reported involvement of GPCRs in numerous non-IgE mediated mast cell activation (79). Altogether, the aforementioned findings suggest ENM potential ability to directly interact with and induce mast cell degranulation, possibly through multiple non-IgE-mediated mechanisms.

It is important to mention that basophils express the FcεR1 receptor and have overlapping properties and functions with mast cells. As such, one may speculate that ENMs that activate mast cells could also lead to similar activation in basophils. Nevertheless, there is limited knowledge regarding ENMs interaction with basophils. It worth noting that only one previous report has established inhibitory properties of fullerene nanoparticles against allergen-mediated stimulation of basophils similar to mast cells (80). Considering future nanomedicine administered through the intravenous route, investigating potential ENM interaction with basophils deemed necessary.

Prior research has shown recruitment of eosinophils (eosinophil flux) to inflammatory sites following pulmonary exposure to different ENMs including metal and metal oxide nanoparticles and carbon nanotubes (81–84). However, to date, there is only limited mechanistic work about direct ENMs interaction with eosinophils. For instance, it has been previously shown in a human eosinophilic cell line (AML14.3D10) that exposure to ZnO nanoparticles and AgNPs resulted in cytoskeletal breakdown and release of inflammatory cytokines as well as induced apoptosis (85). Palladium, which is mainly emitted in the environment from automobile catalytic converters, was previously linked with allergic responses. Recently, it has been demonstrated that exposing AML14.3D10 cells and primary eosinophils isolated from healthy volunteers to palladium nanoparticles (PdNPs), despite lack of direct cellular toxicity, led to adhesion of eosinophils onto endothelial cells through an actin-dependent mechanism (86). These findings suggest a possible ENM-mediated direct interaction with and activation of eosinophils. Nevertheless, confirming these findings in *in vivo* settings is yet to investigated.

## Enm-Induced Suppression of Type I Allergic Reactions

### ENM-Mediated Influence on Antigen Presenting Cells (APCs)

Although all the discussed studies mentioned above demonstrated ENM potential to trigger or worsen outcomes of type I allergic hypersensitivity reactions, there exist studies in the literature demonstrating potential ENM capacity to suppress immune responses. For instance, it has been shown previously that ENM-mediated interaction with APCs may lead to suppression of their function. For example, it has been found that exposure of human monocyte-derived DCs to poly vinylalcohol-coated super-paramagnetic iron oxide nanoparticles (PVA-SPIONs), although was neither associated with changes at the expression level of key surface markers (e.g., CD80 and CD86) nor modulation of antigen uptake, reduced antigen processing, cytokine release and subsequent activation of CD4+ T-cells (87). Similarly, *in vitro* exposure of mouse bone marrow-derived DCs to single-walled carbon nanotubes (SWCNTs) resulted in reduced proliferation of naïve T-cells (88). Furthermore, these findings have been demonstrated *in vivo* following pharyngeal aspiration of the SWCNTs which reduced proliferation of splenic T cells (although there was a local pulmonary inflammation). Altogether, these results suggest that exposure to ENMs could also impair or result in suppressed systemic immune responses, which could potentially influence type I allergic hypersensitivity responses. Indeed, it has been reported previously that *in vivo* exposure to glycine-coated polystyrene nanoparticles resulted in suppressed Th2-type responses following allergen challenge including suppression of lung airway inflammation, airway mucus secretion, serum antigen IgE levels and Th2 type cytokines (89). Interestingly and in accordance with the aforementioned studies, these responses have been found to be mediated potentially via inhibition of DC expansion and subsequent proliferation of allergen-specific CD4+ T-cells (89).

### ENM-Mediated Influence on Th2-Type Effector Cells

Previous literature has also shown that ENM-mediated immunosuppressive properties, beside impacting APC function, they could influence the function of Th2-type effector cells involved in type I allergic hypersensitivity reactions. A number of studies have reported ENM-mediated suppressive responses on mast cells. For instance, in a human mast cell line (HMC-1 cells), ZnO nanoparticles have been shown to inhibit phorbol-myristate-acetate (PMA)/A23187-mediated mast cell release of inflammatory mediators through inhibition of NF-κB, caspase-1, ERK, IKKβ, and RIP2 signaling pathways (90). Importantly, these findings were confirmed *in vivo* in a mouse model of type I allergic hypersensitivity. Specifically, it has been demonstrated that pre-exposure to ZnO nanoparticles orally or topically suppressed allergen-mediated passive cutaneous anaphylaxis (PCA) (90). Another study has shown that fullerenes (carbon Nano spheres) and their derivatives inhibited *in vitro* as well as *in vivo* mast cell activation including attenuation of the inflammatory response and pathological outcomes in experimental mouse models of arthritis and asthma (91–93). In an attempt to understand the underlying molecular mechanisms of the fullerene-mediated inhibition of mast cells, the authors have found that inhibitory properties of fullerenes on mast cell allergen activation is meditated through interference with the FcεR1 signaling.

### Use of ENMs in Allergen Immunotherapy

It is also of relevance to mention in this review that a wide range of ENM compositions (e.g., liposomes, polymers, virus-like particles, etc.) have been utilized among a number of novel therapeutic approaches in allergen immunotherapy, a therapy based on using allergen-specific desensitization approaches to alleviate type I allergic responses. In allergen immunotherapy, ENMs are utilized mostly for targeting/delivery of antigen (e.g., prevent degradation of loaded allergen, enhance uptake by APCs and prevent IgE receptor engagement, co-loading of antigen with immunomodulators, etc.) and/or their tolerogenic/adjuvancy properties (e.g., stimulation of Th1 and/or Treg cells to counterbalance Th2 cells) (94). Indeed, a number of studies have shown the effectiveness and improved therapeutic outcomes when using ENMs for allergen immunotherapy. For instance, in a mouse model of atopic dermatitis (AD) (induced by topical application of 2,4-dinitrofluorobenzene, DNFB), application of hydrocortisone-loaded chitosan nanoparticles transcutaneously have been demonstrated to have superior properties in suppressing pathological manifestations of AD including inflammatory cytokines, IgE and histamine levels, fibroblast infiltration, and anatomical changes at the skin level (95). However, the majority of these studies are primarily concerned with targeted delivery of antigen and it is yet to be determined whether ENMs utilized in such studies could have inherent inhibitory properties. Other studies have utilized ENMs of different compositions and physicochemical properties to target liver antigen presenting cells including Kupffer cells and liver sinusoidal endothelial cells and have demonstrated tolerogenic capacity. This could indeed open a new avenue in the treatment of hypersensitivity conditions such as type I allergic responses and autoimmune diseases (vs. only relieving the symptoms). For instance, it has been recently shown in a mouse model of ovalbumin-induced airway allergic disease that ovalbumin-loaded poly(lactic-co-glycolic acid) (PLGA) nanoparticles decorated with mannan (targeting mannose receptors) or ApoB (targeting scavenger receptors) for targeting liver sinusoidal endothelial cells were capable of stimulating Tregs resulting in systemic immune tolerance and alleviating disease outcomes (96). Therefore, ENMs should not always be negatively associated with allergic responses. Although evidence of inherent inhibitory properties of ENMs is currently limited, it would not be surprising to unravel ENMs with such properties in the future. Nevertheless, thorough characterization of ENM physicochemical properties and further understanding of ENM structure-activity (immunomodulatory properties) relationship as well as the underlying molecular mechanisms are key for exploiting nanomaterials toward novel therapeutic applications.

## Conclusions

Materials scientists and chemists are engineering nanomaterials with atomic precision. Indeed, the advent of nanotechnology and the ability to fabricate and manipulate matter at the atomic and molecular levels has led to a diverse range of nanomaterials and ever emerging ENMs with novel physical, chemical, and biological properties. As a result, one of the major challenges in assessing the safety of nanomaterials is the existence of a diverse range of compositions (e.g., metals, carbon-based, polymers, lipid-based, a composite of materials, etc.) and physicochemical properties (e.g., size/shape, surface morphology and functionalization, stability in physiological media, etc.). This makes carrying out comprehensive *in vitro* and *in vivo* research to assess ENM allergenicity for each of these diverse nanomaterials not practically feasible. Therefore, establishing new, reliable, high throughput methods for screening ENMs for potential allergenicity is highly recommended. Indeed, use of alternative approaches (e.g., use of *in vitro* human models, computational modeling, etc.) in the assessing ENM toxicity in general have been previously proposed (97–99). Furthermore, it would be more reasonable to carry out further detailed investigations on candidate ENMs that show no potential allergenicity on the preliminary screening assays. Establishing such reliable methods could save the research community as well as the pharmaceutical/biotechnology industry huge financial and labor burdens, which could be utilized toward other important aspects of nanosafety research.

It's worth mentioning that one of the important research outcomes over the past few years in the area of ENM-induced immunomodulation particularly within the context of type I hypersensitivity is that even those ENMs that are considered inert and safe under certain conditions can be problematic. This finding underscores the importance of risk assessment of those ENMs that lack major toxicity in contexts of relevance and in real case scenarios including those of type I allergic hypersensitivity responses.

It is also important to bear in mind that metals and metal oxides represent a large subgroup of ENMs that have numerous applications and wide industrial use. Metals have long been associated with various immune hypersensitivity reactions including type I allergic responses (100). Indeed, a number of the discussed studies in this review, which have been shown to be associated with type I hypersensitivity reactions, were ENMs of metal and metal oxide origin. This topic remains one of the research areas that is largely understudied, particularly, the underlying molecular mechanisms associated with ENM-induced allergic and allergic-like responses. For further information on the topic, an excellent, comprehensive review has been recently published about ENMs of metal origin and allergic disease (101).

Last but not least, it is also important to note that it would be almost impossible to rank ENMs or generalize into major categories in terms of ENM potential allergenicity in the context with type I hypersensitivity given the limited available literature on the topic as well as the diverse nature of ENMs. Therefore, establishing reliable methods to screen ENMs for potential allergenicity could bridge this gap and points out specific materials/compositions and physicochemical properties that are likely associated with type I allergic responses. Such findings could also pave the road for utilizing a “safety-by-design” approach for engineering future nanomaterials that are devoid of major type I hypersensitivity reactions.

In summary, accumulating evidence indicates that ENMs, of a wide range of compositions and physicochemical properties, are capable of triggering and/or exaggerating/priming immune responses toward type I hypersensitivity reactions. Although the underlying molecular mechanisms remain largely unknown, prior research suggests that multiple mechanisms could explain at least partially how ENMs might influence type I allergic hypersensitivity. This includes (i) ENM-mediated direct interaction with pattern recognition receptors and/or release of alarmin molecules which results in the activation of the inflammasome; (ii) direct interaction with and activation of the complement system either through ENM inherent properties or acquired properties due to formed biocorona; (iii) direct interaction with APCs (activation or suppression) including modulation of antigen processing and presentation; and finally, (iv) direct interaction with and activation of key Th2-type effector cells such as mast cell, basophils and eosinophils leading to immediate degranulation and release of vasoactive molecules. Beside establishing new high-throughput type functional methods that evaluate potential allergenicity of ENMs, future studies assessing type I hypersensitivity should also address other key aspects including (i) thorough characterization of ENM physical and chemical properties including stability over time and formation of ENM biocorona in relevant biological media; (ii) identification of key structure-activity relationship (SAR); (iii) elucidation of common molecular pathways of exposure/disease; and finally, (iv) development of representative preclinical animal models for type I allergic hypersensitivity.

## Author Contributions

NA wrote the manuscript. JB conceived of and edited the manuscript.

### Conflict of Interest

The authors declare that the research was conducted in the absence of any commercial or financial relationships that could be construed as a potential conflict of interest.
